# Unsuccessful Progression of Sacral Neuromodulation From the Evaluation to the Implantation Stage: A Single-Center Experience

**DOI:** 10.7759/cureus.16912

**Published:** 2021-08-05

**Authors:** Mohammad A Alghafees, Mohammed K Alageel, Meshari A Alqahtani, Yahia Alghazwani

**Affiliations:** 1 College of Medicine, King Saud Bin Abdulaziz University for Health Sciences, Riyadh, SAU; 2 Urology, King Abdulaziz Medical City, Riyadh, SAU

**Keywords:** urology, sacral neuromodulation, bladder dysfunction, neuro-urology, saudi arabia

## Abstract

Introduction

Over the years, sacral neuromodulation (SNM) has become an established and effective treatment for chronic urinary system retention and incontinence. The process of SNM is performed in two stages, the first is an evaluation phase and the second an implant phase. This study aimed to assess the rate of failure of progression from the evaluation to the implantation stage and the factors predicting the outcome of this commonplace procedure.

Materials and methods

This retrospective cross-sectional study took place at King Abdulaziz Medical City (KAMC), Riyadh, Saudi Arabia. All the patients who underwent SNM implantation from January 1, 2016 to January 1, 2021 were included. Patients younger than 14 years and patients who had the SNM implantation in a different hospital and were only followed-up at KAMC were excluded. Patient-related information were extracted from the BESTCare system. Frequency and percentage were used for the categorical variables, and the mean, median, and standard deviation to display the continuous variables. Chi-square test and Mann-Whitney U test were used to test for the association of the categorical variables.

Results

Among 28 patients, 46.4% (n=13) failed to progress from the evaluation phase to the implantation phase. Gender, age, having a co-morbidity, and SNM indication were not significant factors for predicting the outcome of the SNM evaluation phase.

Conclusion

The observed failure rate was marginally higher than the ones detected in other studies. Although no significant association was detected between evaluation stage failure and the assigned predictors, the results need to be interpreted with caution due to the small population size. Larger multicenter studies need to be done in order to investigate the link between patient characteristics and the efficacy of SNM. Establishing a concrete evidence would further refine the targeted patient population and indications for SNM.

## Introduction

Over the years, sacral neuromodulation (SNM) has become an established and effective treatment for chronic urinary system retention and incontinence. The process of sacral neuromodulation is performed in two stages, the first is an evaluation phase and the second an implant phase. In the evaluation phase, the physician assesses whether or not the symptoms will be adequately treated with SNM. Also, it gives a chance for the patient to modify their lifestyle and daily life with the SNM device implanted. On average, the evaluation phase takes between two to eight weeks [1]. In some instances, the evaluation phase could fail due to many reasons, such as infection, breaking of lead, or patient dissatisfaction [2]. However, there is emerging evidence that other parameters, such as age, gender, SNM indication, and body mass index (BMI) could have a role in increasing the likelihood of failure to progress to the implantation stage. 

In Saudi Arabia, with the increasing use of SNM, urologists are more likely to encounter evaluation stage failures, the presented study aimed to assess the rate of failure of progression from the evaluation to the implantation stage and its predictors among patients undergoing sacral neuromodulation in King Abdulaziz Medical City (KAMC), a tertiary academic hospital in Riyadh, Saudi Arabia. Further studies in the region exploring the failure of SNM treatment among patients would further refine the targeted patient population and indications for SNM consequently improving patient outcomes.

## Materials and methods

This retrospective cross-sectional study was conducted at KAMC, Riyadh, Saudi Arabia. All the patients who underwent SNM implantation from January 1, 2016 to January 1, 2021 were included. Patients younger than 14 years and patients who had the SNM implantation in a different hospital and were only followed-up at KAMC were excluded.

Demographic information, comorbidities, the indication for SNM, and failure at the evaluation stage of implantation were extracted from the BESTCare system (ezCareTech, South Korea). The data were entered in Microsoft Excel 2019 (Microsoft Corporation, WA, USA) and the statistical analysis was done with the Statistical Package for the Social Sciences (SPSS) version 23.0 (IBM Corporation, Armonk, NY, USA). Frequency and percentage are used to display the categorical variables, and the mean, median, and standard deviation to display the continuous variables. Chi-square test and Mann-Whitney U-test were used to test for association of gender, age, co-morbidity presence, and SNM indication with the outcome of the evaluation stage in SNM. The level of significance was set at 0.05.

The patients' confidentiality and anonymity were ensured, as serial numbers replaced the medical record numbers. The data was accessed and used by only the research team. The Institutional Review Board of King Abdullah International Medical Research Center, the Ministry of National Guard-Health Affairs, Riyadh, Kingdom of Saudi Arabia, approved the study with approval number NRC21R/095/03.

## Results

The final population size was 28 patients. Table 1 displays the sociodemographic profile of the sample. Of the population, 46.4% (n=13) were males and 53.6% (n=15) were females. The minimum age was 17 years, the maximum was 73 years, and the mean age was 37.14 + 14.62 years.

**Table 1:** Socio-demographic profile of the patients (n =28).

Demographical characteristic	n	%
Gender		
Male	13	46.4
Female	15	53.6
Age		
Mean	37.14	
Standard deviation	14.62	
Minimum	17	
Maximum	73	

**Figure 1:**
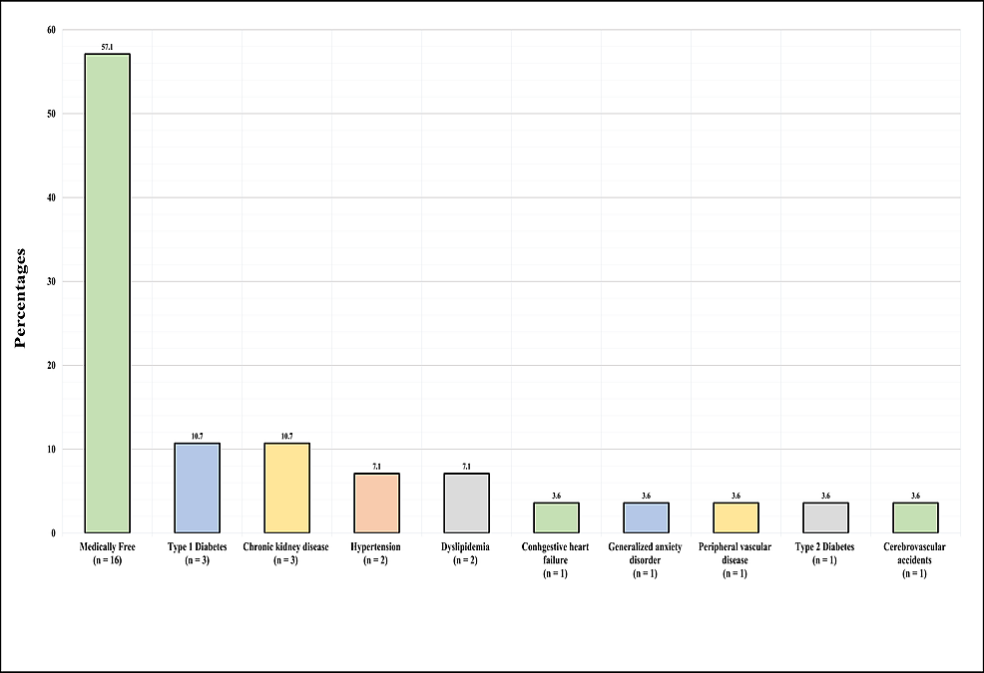
Presence of comorbidities among participants.

Figure 1 displays the co-morbidities occurring in the sample. More than half (n=16, 57.1%) were medically fit, 10.7% (n=3) had type 1 diabetes, 10.7% (n=3) chronic kidney disease, 7.1% (n=2) had hypertension, 7.1% (n=2) had dyslipidemia, 3.6% (n=1) congestive heart failure, 3.6% (n=1) generalized anxiety disorder, 3.6% (n=1) had peripheral vascular disease, 3.6% (n=1) had type 2 diabetes, and 3.6% (n=1) had a cerebrovascular accident.

**Figure 2:**
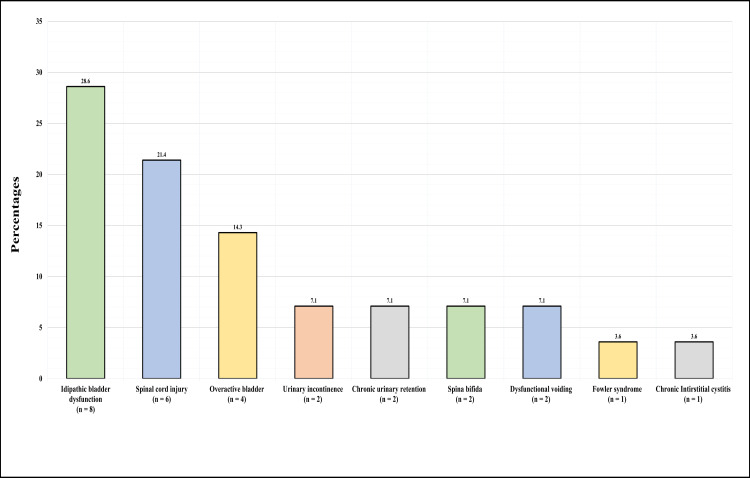
Primary diagnoses among participants.

Figure 2 presents the patients’ diagnoses. The highest proportion, 28.6% (n=8), had an idiopathic bladder dysfunction, 21.4% (n=6) had a neurogenic bladder due to a spinal cord injury, 14.3% (n=4) had an overactive bladder, 7.1% (n=2) had urinary incontinence, 7.1% (n=2) had chronic urinary retention, 7.1% (n=2) had spina bifida, 7.1% (n=2) had dysfunctional voiding, 3.6% (n=1) had Fowler syndrome, and 3.6% (n=1) had chronic interstitial cystitis.

Table 2 demonstrates the trials done with the patients and the outcome. 46.4% (n=13) Failed to progress from the evaluation phase to the implantation phase.

**Table 2:** Outcome of SNM trials and implantations. SNM: sacral neuromodulation.

	n	%
Evaluation phase		
Successful	15	53.6
Unsuccessful	13	46.4
Implantation phase		
Not done	16	57.1
Successful	12	42.9

Table 3 displays the factors associated with failure in the evaluation stage. No significant association was found between failure in the evaluation stage and gender, age, having a co-morbidity, and SNM indication.

**Table 3:** Factors associated with failure at evaluation stage.

Predictor	Evaluation trial outcome		p-value
	Success	Failure	
Gender			0.464
Male	6 (46.2%)	7 (53.8%)	
Female	9 (60%)	6 (40%)	
Age	31.93 + 11.81	43.15 + 15.67	0.065
Presence of a comorbidity			0.743
No	9 (56.3%)	7 (43.8%)	
Yes	6 (50%)	6 (50%)	
Indication			0.055
Spinal cord condition	2 (25%)	6 (75%)	
Bladder condition	13 (65%)	7 (35%)	
*Significant at level 0.05			

## Discussion

There is emerging evidence that other parameters, such as age, gender, SNM indication, and body mass index (BMI) could have a role in increasing the likelihood of failure to progress to the implantation stage. For instance, unlike our study which found no link between SNM indication and trial outcomes, a Turkish study evaluating the progression rates from the evaluation phase to the implantation phase stated that all the patients who failed to progress had an underlying diagnosis of an overactive bladder [3]. In the United States, a similar report has emerged stating a link between BMI and evaluation stage failure. The study also identified a higher chance of evaluation phase success among females compared to males which also contradicts our negative findings. Moreover, patients with an urgency incontinence had higher chances of evaluation phase success compared to patients with urgency-frequency [4].

Nowadays, sacral neuromodulation is widely used for the treatment of many diseases which include but are not limited to overactive bladder, urinary retention, and intractable constipation [5,6]. The SNM implantation usually occurs in two stages with testing for the symptoms between them [7]. In our study, we found that 53,6% of our patients had success in the evaluation stage and went on to the implantation stage. Compared to our results, other studies showed marginally higher success rates ranging between 76% and 93.2% [8-14].

In regard to the predictors of success of the evaluation stage, we found that there is no sufficient link between SNM evaluation success and age, gender, comorbidities, or indication which is similar to a Dutch single-center experience which concluded that neither gender, patient age, history nor diagnosis were predictors of evaluation stage success [15]. Conversely, many other studies found that women were more likely to experience success at the evaluation stage and proceed to the implant stage [4,16,17]. Also, other studies found an association between age and success of SNM evaluation with younger patients having a better response to SNM than older patients [18,19]. Furthermore, some studies found that patients who had urge urinary incontinence as the indication for sacral neuromodulation showed a significantly higher success rate [20].

The presented study had some limitations. First, the retrospective study design is one of our limitations. We could only look at data that had been previously entered into the patients' medical records. Consequently, we had to rely on others for accurate and safe record keeping. Second, the population size of the study is smaller than other larger multicenter studies done globally which may not be representative of the whole SNM population in Saudi Arabia. Hence why further prospective, randomized, multicenter studies are needed to assess the failure rate in the evaluation stage of sacral neuromodulation and the predictors of success since there is a discrepancy in the studies that we found in the literature.

## Conclusions

The observed failure rate was marginally higher than the ones detected globally. In contrast to previous studies, no sufficient link has been observed between failure in the evaluation stage and gender, age, having a co-morbidity, or SNM indication. Sacral neuromodulation is shown to be an advanced treatment of some chronic urinary system diseases. However, further studies are needed to establish a reliable result to improve the use of SNM and further refine its indication criteria in order to achieve the optimal results, especially in the region, i.e., Saudi Arabia. The magnitude of the effect comorbidities have on SNM can be better explained with the use of a performance status criteria, such as ECOG and NYHA FC, which the presented study failed to do so due to its small population size. Additionally, factors that could affect the subjective perception to the quality of therapy, such as the concomitant depression and anxiety in patients with bladder disorders need to be further explored as they might affect the patients' satisfaction during the evaluation phase, there­by improving patients' quality of life.
